# Nanometer-resolved mechanical properties around GaN crystal surface steps

**DOI:** 10.3762/bjnano.5.225

**Published:** 2014-11-19

**Authors:** Jörg Buchwald, Marina Sarmanova, Bernd Rauschenbach, Stefan G Mayr

**Affiliations:** 1 Leibniz-Institut für Oberflächenmodifizierung e.V. (IOM), Permoserstr. 15, 04318 Leipzig, Germany; 2 Fakultät für Physik und Geowissenschaften, Universität Leipzig, 04103 Leipzig, Germany; 3 Translationszentrum für regenerative Medizin (TRM), Universität Leipzig, 04103 Leipzig, Germany

**Keywords:** finite elements, gallium nitride, indentation, mechanical properties, molecular dynamics, nanostructures

## Abstract

The mechanical properties of surfaces and nanostructures deviate from their bulk counterparts due to surface stress and reduced dimensionality. Experimental indentation-based techniques present the challenge of measuring these effects, while avoiding artifacts caused by the measurement technique itself. We performed a molecular dynamics study to investigate the mechanical properties of a GaN step of only a few lattice constants step height and scrutinized its applicability to indentation experiments using a finite element approach (FEM). We show that the breakdown of half-space symmetry leads to an “artificial” reduction of the elastic properties of comparable lateral dimensions which overlays the effect of surface stress. Contact resonance atomic force microscopy (CR-AFM) was used to compare the simulation results with experiments.

## Introduction

Recently developed scanning probe-based techniques, such as contact resonance atomic force microscopy (CR-AFM) [1–2], allow for the assessment of mechanical properties of soft and hard condensed matter surfaces at the nanoscale. Such developments make it even more compelling to look at nanometer-sized materials from a theoretical point of view to gain a deeper understanding of the physical mechanisms involved in mechanical response. Several studies have focused on the mechanical properties of nanostructures such as nanowires [3–4] or nano-sized granular films [5] by using indentation methods. Here, generally two major challenges arise. On the one hand, the varying tip–surface contact area has to be taken into account. On the other hand, the breakdown of half-space symmetry hinders a straightforward analysis, especially when stressfields significantly exceed the sample dimensions or become severely influenced by domain boundaries. Therefore, in the present study, a simple step of several atomic layers height has been studied as a model system in order to gain insight into the relevant physics and/or artifacts caused by a specific measurement technique.

While it is established that surfaces lead to an effective change of the elastic constants, e.g., a reduction due to surface stresses within a thin film [6], mechanical properties around more advanced surface features, including steps, are unclear at this point. The present work addresses the mechanical behavior around a gallium nitride (GaN) step employing a combination of classical molecular dynamics (MD) simulations with a finite element (FEM) approach and CR-AFM experiments. GaN is a material of great interest due to its application as a wide-band-gap semiconductor especially for optoelectronic devices [7–8]. Additionally, it is known that GaN can exhibit terraces ranging from monoatomic to a few lattice parameters in step height [9–10]. In principle, the results of this paper can be generalized, due to the fact that they are based on a general formalism introducing surface stresses in continuum mechanics and since many other materials ranging from ionic crystals [11] to metals [12] are known to form such steps.

## Theoretical considerations

A step can be described by the set

[1]
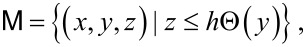


where *h* is the step height and Θ the heaviside step function. This paper will focus on how the surface stress at *y* = 0 influences the mechanical properties at a given distance on the top and at the bottom of the step (close to the surface). Therefore, the surface stress of the planes {(*x*,*y*,*h*) | *y* ≥ 0} and {(*x*,*y*,0) | *y* ≤ 0} will be neglected as it is constant along *x* and *y*, but its treatment would follow the same procedure concerning surface elasticity and symmetry arguments as depicted in the following approach.

We first sub-divide the material of interest into infinitesimal cubes of the volume *dV*. The resulting force acting on a partial volume is equal to the sum of all forces acting on each single element. The divergence theorem connects the force acting on a volume element with the stresses at its boundaries:

[2]
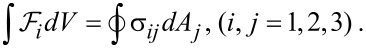


The force (expressed in terms of force per unit volume 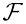) acting on volume element *dV* at point *p* arises from the effective stresses acting on the boundaries of its neighboring volumes. Due to the fact that the tensile stresses are homogeneous along {(*x*,0,*z*) | 0 ≤ *z* ≤ *h*}, the near field behavior of the stress field is a reduction just along its lateral distance *y* from the step edge. This fact can be expressed by considering the surface stress, which is the variation of the total surface energy as a function of the strain, *ε*:

[3]
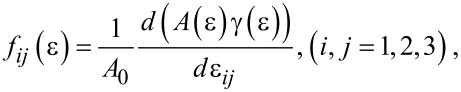


where *A*_0_ is the surface area before deformation and *γ* the surface energy. Therefore, the local stresses σ*_ij_*(*y_p_*) which are acting on the boundaries of *dV* can be written in terms of the surface stress *f_ij_*:

[4]
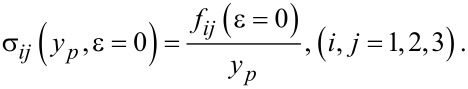


By defining the constants *c_ijkl_*(*ε*) *ε_kl_* := *f_ij_*(*ε*) − *f_ij_*(*ε* = 0) and applying Hooke’s law, it is possible to calculate local effective elastic constants as a function of their distance *y_p_* from the step edge:

[5]
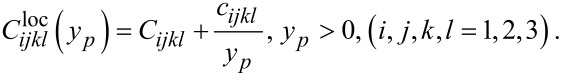


Analogously, it is also possible to understand the behavior below the step, i.e., for *y* < 0. The stress induced by the step does not only decrease along −*y*, but also along −*z* due to the fact that from this point of view, the stresses arising from the step are homogeneous along the x-axis only. This can be also expressed by a line tension *F_i_* along the step edge, which defines the non-zero elements of the edge elastic tensor *d_iijj_*(*ε*)*ε_jj_* := *F_i_*(*ε*) − *F_i_*(*ε* = 0). As a consequence of the line tension, the stressfield is spread cylindrically from its origin along −*z* and −*y*. Therefore, the following relation for the elastic constants is obtained:

[6]
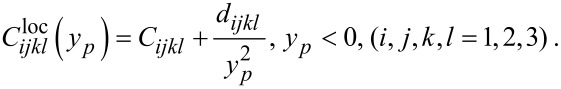


From linear response theory, it is also possible to derive a microscopic expression for the elastic constants [13]:

[7]
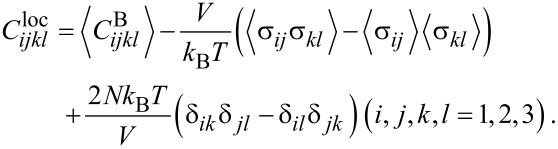


The last (kinetic) term can be omitted at low temperatures. The Born-term 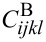 = 1/*V∂*^2^*U*/(*∂ε_ij_∂ε_kl_*) will just differ for edge atoms, therefore the stress-induced effective change in the elastic properties along *y* will essentially lead to a change in the stress-fluctuation term. Equation 7 allows us to study the mechanical response at a step by means of MD simulations. Here, the explicit calculation of the elastic constants will be omitted, since the volume per atom *V* is not uniquely defined in any case. Therefore, these expressions have been used to provide a qualitative understanding of the problem and have been accompanied by explicit indentation simulations to quantitatively describe the elastic response of a step.

## Simulation methods

**Figure 1:**
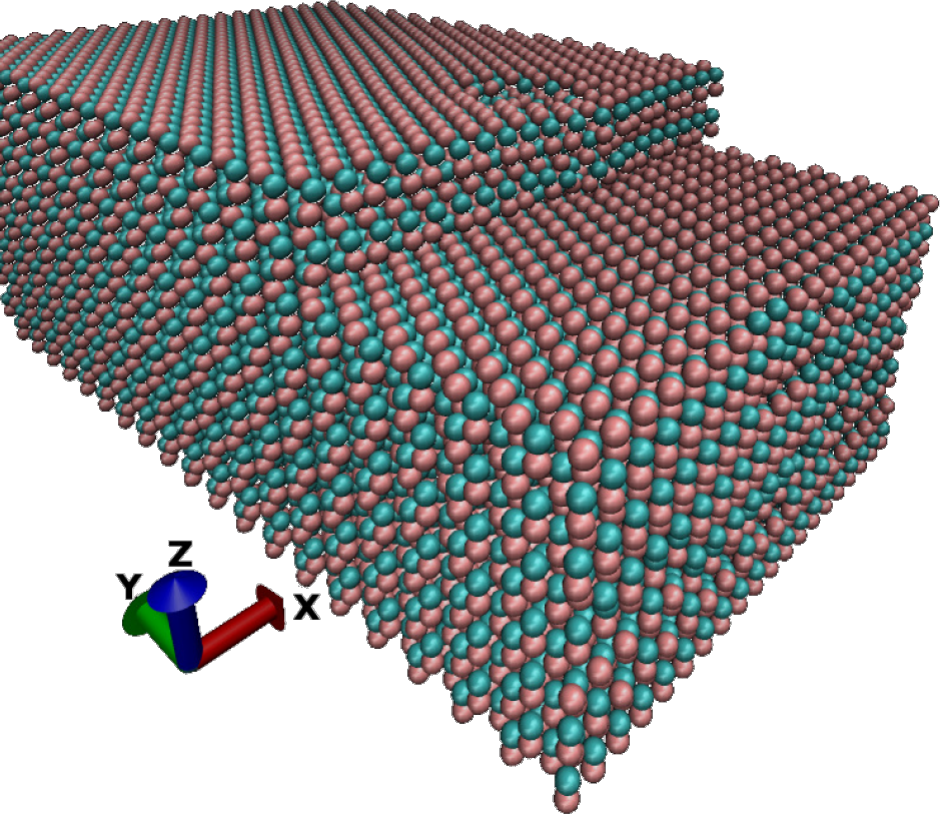
MD configuration of a GaN step with a step height of *h* = 2*c* during indentation of surface atoms within a circular area of *A*_2_ = 1.232 nm^2^. The red spheres denote the gallium and blue spheres the nitrogen atoms.

All MD runs were carried out with the LAMMPS code [14]. The GaN step was modeled in the wurtzite crystal structure oriented along the [001] direction by means of a Stillinger–Weber potential [15]. The simulations were carried out in a NVT ensemble utilizing Nosé–Hoover thermostatting at 5 K [16–17], Verlet time integration at constant volume and free boundary conditions along all three axes. We simulated three different step heights: *h* = 1*c*, 2*c*, and 3*c*, where *c* = 0.521 nm is the lattice parameter of the wurtzite structure along the *z*-direction. For the fluctuation method, 10,000 atoms were simulated over a time period of 100 ps. For the indentation experiments, approximately 70,000 atoms were used with a simulation time period of 100 ps as well. In Figure 1, one sees the MD configuration of a GaN step of height *h* = 2*c*.

The FEM simulations were performed by using the COMSOL software package [18] and experimental elastic constants [19]. The model domain was 120 nm × 120 nm × 80 nm large with free boundaries along *x* and *y*, with a fixed boundary as the substrate. The tetrahedral mesh consisted of approximately 180,000 elements, including extra refinements at the contact region.

## Results and Discussion

### Simulation Results

**Figure 2:**
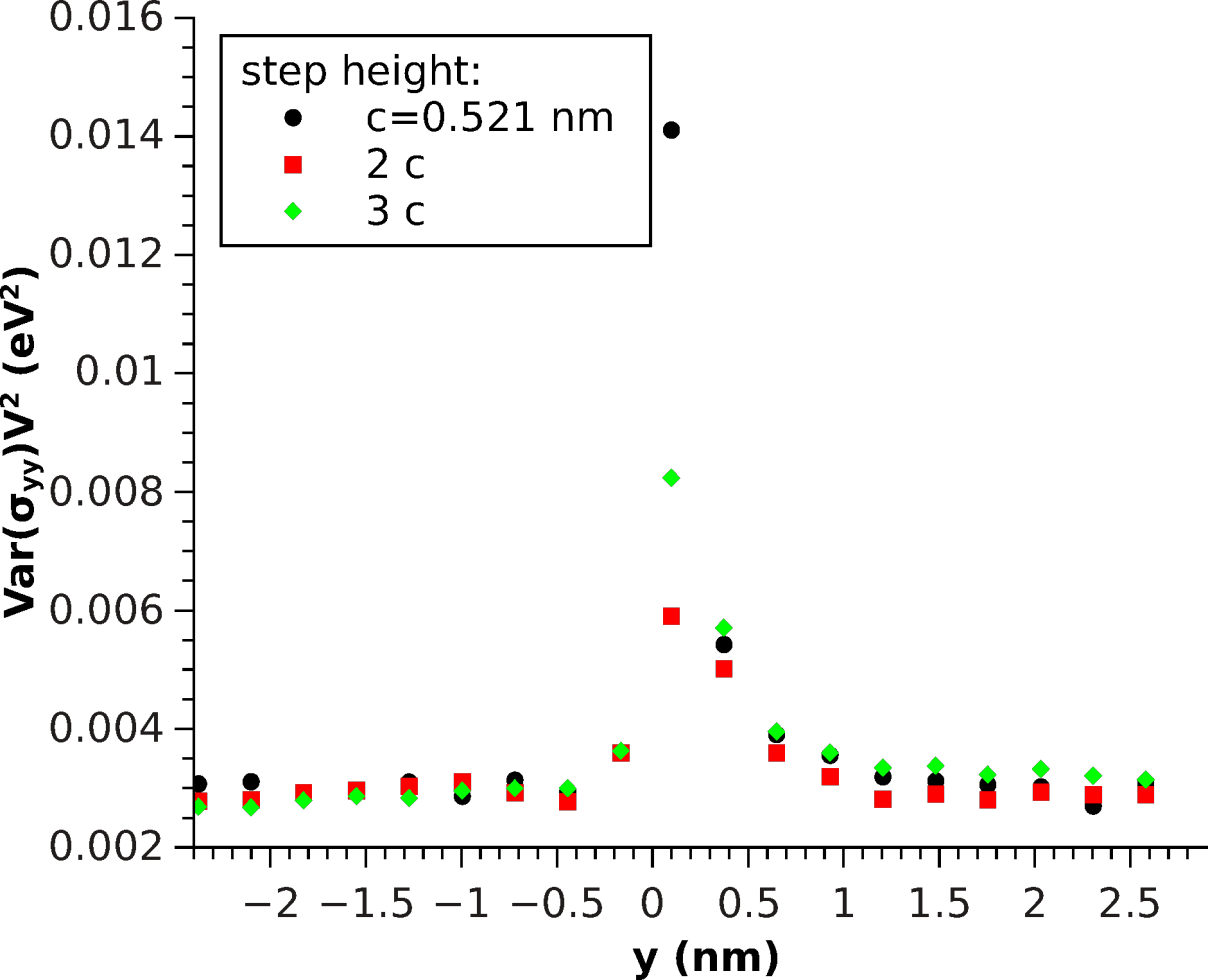
Stress fluctuation multiplied by the squared per-atom volume 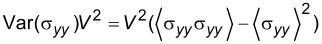 along the y-axis of the upper Ga atoms.

In Figure 2, the *yy*-component of the stress variance, representing *C_yyyy_*, is given in squared energy units due to the fact that the per-atom volume for surface and edge atoms, in particular, is not well defined. At the top of the step, i.e., for *y* > 0 one observes the expected 1/*y* behavior (Equation 5), which is comparable for all step heights due to nearly identical surface stresses, with exception of the first atom particularly for the smallest step height. This can easily be understood by taking into account the interaction of all three surfaces and differences in the per-atom volume at this point. Below the step, the stress fluctuations are nearly constant for all step heights except for the closest atom which feels the stress significantly. This behavior reflects the better convergence properties of 1/*y*^2^ (Equation 6).

From an experimental point of view, it is hardly possible to evaluate the microscopical expressions (Equation 7) for the elastic constants. Therefore, the mechanical properties were investigated in terms of the indentation (or reduced Young’s) modulus *M* by using an indenter acting with a load *F* on a contact area *A* of a half-space, thereby causing a displacement *u* [20]:

[8]
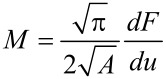


Since the elastic constants can consistently be extrapolated on an atomistic level (Equation 7), we also expect *M* to give reasonable values at this scale. For simulating a flatpunch indentation, forces of about 10–30 eV/nm were applied directly to each gallium surface atom within a certain radius *r*_c_. As contact area *A*, the product of the two-dimensional Wigner–Seitz volume and the number of surface atoms within that radius were chosen. The indentation simulations were carried out by using approximately 70,000 atoms on a fixed substrate and free boundaries along the other sides.

**Figure 3:**
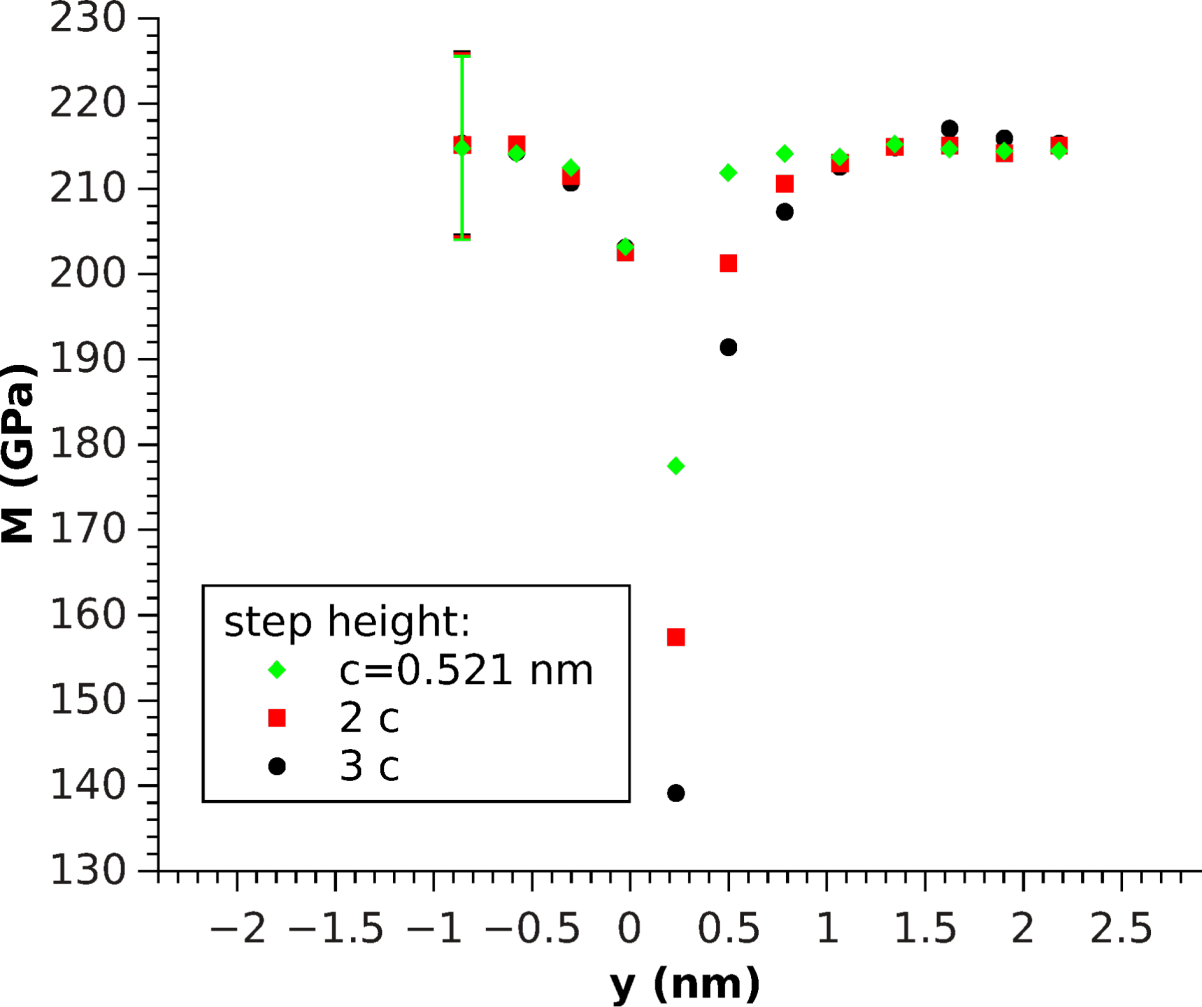
Local [001]-oriented indentation modulus for three different step heights along the *y*-axis. Measured by indenting a single atom (*A*_1_ = 0.088 nm^2^). The error bars denote the standard deviation of the displacement and apply to all data points.

**Figure 4:**
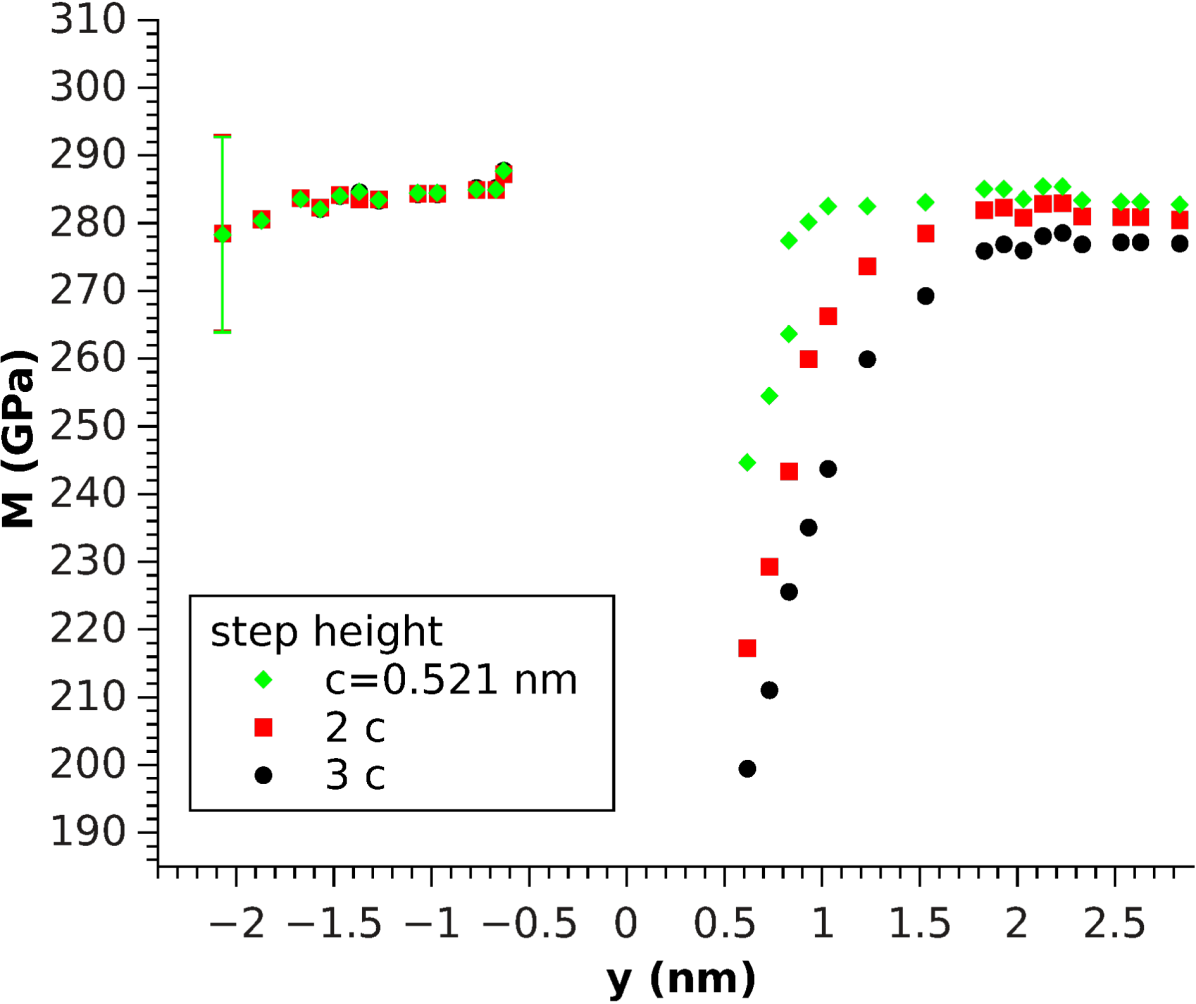
[001]-oriented indentation modulus for three different step heights along *y*. Measured by indenting a circular region of surface atoms (*A*_2_ = 1.232 nm^2^). The error bars denote the standard deviation of the displacement and apply to all data points.

The step was investigated by using two contact areas: *A*_1_ = 0.088 nm^2^ and *A*_2_ = 1.232 nm^2^ which correspond to one and 14 indented atoms, respectively (Figure 3 and Figure 4). The asymptotic (far from *y* = 0) elastic constants differ for both cases due to the difference in contact area. This difference leads to a change in surface sensitivity and affect the influence of the boundary conditions, which play a more pronounced role for greater contact radii due to the increased extension of the stress field. Under these conditions both values are in reasonable agreement with the experimental value of *M*_lit_ = 330 GPa, which was calculated from the elastic constants [19] by using the formalism presented by Vlassak and Nix [20], who connected the indentation modulus to the Barnett–Lothe tensors for anisotropic materials [21].

For the one-atom indentation, (Figure 3), *M* behaves similarly to stress fluctuations with better convergence below the step and a similar decrease in the elastic constants on top, approaching the step edge, comparable to the theoretical predictions (Equation 6 and Equation 5) for the elastic constants. Therefore, it can be concluded that one-atom indentation describes the stress distribution quite well, except for the fact that a clear (monotone) difference between different step heights was observed. Indeed, the step seems to soften with increasing step height. This trend is even more pronounced for the greater contact radius (Figure 4). Another matter that is conspicuous in this case is the fact that the softening for *y* < 0 is not measurable anymore, rather, a tiny increase for the smallest distance is observed, independent of the step height. Even on these scales, both facts indicate that the breakdown of half-space symmetry at *y* = 0 plays a non-negligible role.

**Figure 5:**
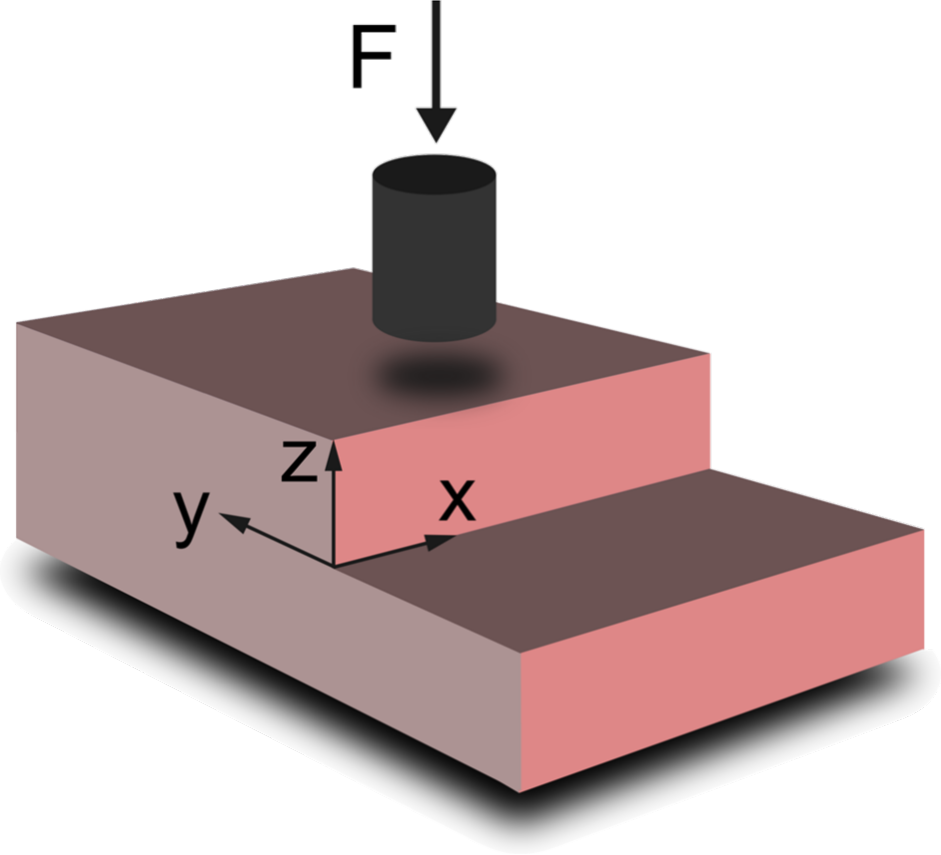
Schematic FEM configuration of the indentation on top of the step by using a flatpunch indenter.

To estimate the influence of this effect, the same contact radius was used to simulate the indentation in a FEM model (Figure 5) in which surface stresses were omitted. The indentation modulus was calculated by evaluating the contact stiffness *S* = *dF*/*du* of the stationary solution. The flatpunch indenter was modeled by a cylinder of hard material with a contact area *A*_2_ and a force of |**F**|= −*F_z_* = 30 nN as an initial condition.

**Figure 6:**
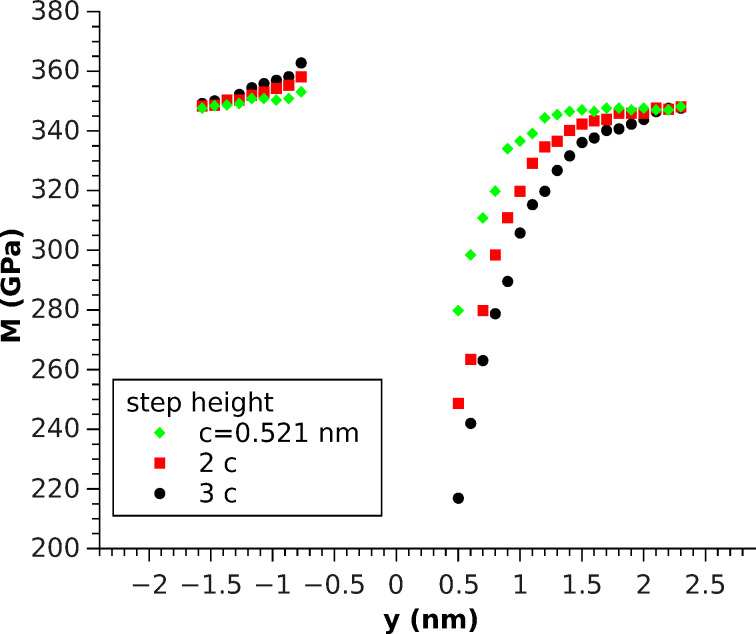
FEM simulation of indentation modulus for three different step heights by using a flatpunch indenter (*A*_2_ = 1.232 nm^2^).

As a result, a distinctive step height-dependent increase for *y* < 0 and decrease (*y* > 0 towards the step) were seen, which could be explained by the outreach of the stressfields induced by the indenter over the region that could be treated as a half-space (Figure 6). Comparing these results to the molecular dynamics simulation with the same radius (Figure 4), a similar behavior for the indentation modulus could be found. However, there was an even more pronounced increase for *y* < 0 that was also dependent on the step height, in contrast to our MD simulations results. Nevertheless, the stress-induced softening, observed when using one-atom indentation, and the hardening, observed in the FEM simulation, seem to nearly compensate in the MD simulation for this contact radius (Figure 4). The decrease of *M* for *y* > 0 towards the step looks quite similar in Figure 4 and Figure 6, which indicates that it plays a dominant role in Figure 4 in addition to the effects caused by the role of stress.

The contact area of *A*_2_ corresponds to a radius of *r*_2_ = 0.626 nm, so there are three moduli *M*(0 < *y* < 0.626 nm) in Figure 6, for which both the stress field in the sample and the contact area are shrinking as *y* approaches 0, due to the size of the contact area of the indenter. However, in this case, the change in the contact area manifests in a small bend and appears to play a minor role.

### Experimental Results

In order to validate the theoretical results, CR-AFM studies on GaN thin films were undertaken. CR-AFM is a technique for evaluating the mechanical properties of a broad range of materials while using one of the highest lateral resolution available, compared to other recent methods [1–2,22–23]. The investigated sample was an epitaxial c-plane GaN film grown on a 6H-SiC substrate by ion beam-assisted molecular beam epitaxy [24]. Measurements for the elastic properties of the GaN film were performed by a CR-AFM, that was custom-built into a commercial Asylum Research MFP-3D AFM [25]. The AFM probe used for CR-AFM imaging was a Si PPP-NCLR (NanoSensors, Switzerland) with a spring constant of 39 N/m. The second resonance mode was used for further analysis. The reduced Young’s modulus was measured by using a reference approach with three reference samples: fused silica (*M* = 75 GPa), silicon (*M* = 165 GPa) and sapphire (*M* = 433 GPa), which were demonstrated to be sufficient for quantitative mechanical analysis [26]. From this data, we obtained *M* = 285 GPa with an uncertainty of 5–10%, typical for CR-AFM measurements.

According to previous investigations, the radius of curvature, *R*, of the Si-tip hardly remains below 25 nm during elasticity measurements of stiff materials [26]. Considering a spherical contact and a force loading *F* in the range of 400–500 nN, the resulting contact radius *r*_c_ is determined by [22]

[9]
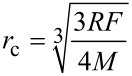


and will be within 4.0–4.5 nm for GaN films. Here, *M* is the indention modulus of the contact that is given by

[10]
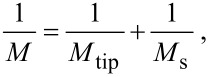


where *M*_tip_ and *M*_s_ are the indentation moduli of the tip and the sample, respectively. Since the influence of the step edge only spreads within a distance of several nanometers from the step edge, the measurements should be performed directly on the step. Therefore, the reduction of the contact area when the tip passes the step edge must be taken into account. When the center of symmetry of the tip passes the step edge, the contact area adopts half of its initial value. Therefore, the contact radius can be estimated to be roughly √2 times less than that of a vibrating tip far from the step. From Equation 8, it is obvious that this decrease in contact area leads to an underestimation of the elastic modulus when maintaining a load.

**Figure 7:**
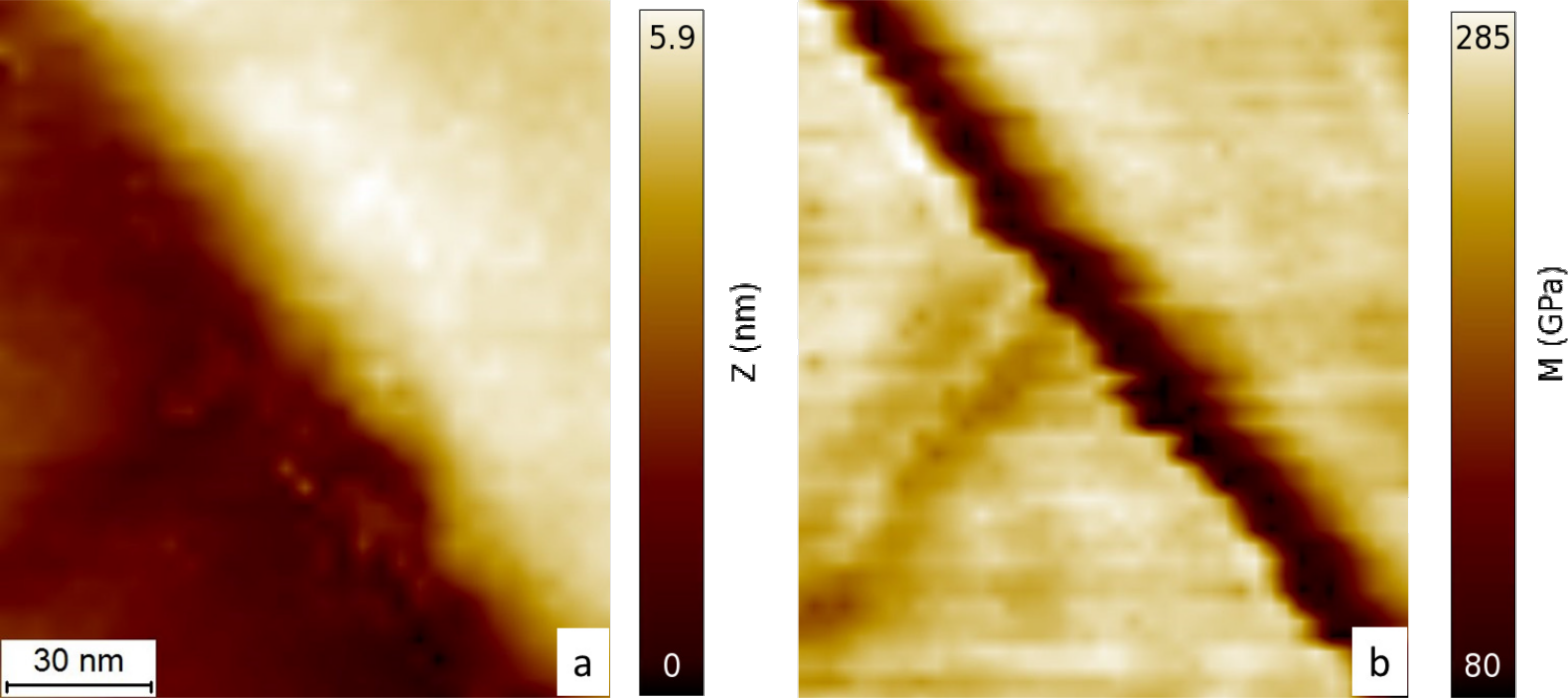
Topography (a) and indentation modulus (b) map of the area around a GaN step.

This effect is demonstrated for a GaN step of 5 nm in height (see Figure 7). The step is high enough to theoretically exhibit a visible reduction in the indentation modulus with a sufficiently sharp tip. Simultaneously, with continuous measurements of the contact resonance frequencies by CR-AFM, the topography image was obtained. When comparing both, the frequency and topography images, a small reduction of resonance frequency was detected near the step edge. Taking into account the diminished contact area compared to flat surface contact, calculation of the indentation modulus by using the reduced contact area leads to the same value as far from the step within accuracy of 10%. At the bottom of the step, where one has to take multiple contact areas into account due to the spherical tip, it was not possible to measure any change. Obviously, the radius of the tip was too large to observe any stress dependence in the indentation modulus. One way to solve this problem is through the use of custom-designed probes with much higher stiffness in order to reduce contact area, making it possible to obtain more accurate elasticity values and thereby detecting the reduction in the indentation modulus with nanometer-sized steps.

## Conclusion

Tensile stresses lead to a significant local effective reduction in the elastic constants. In the case for a step of several nanometers in height, this effect can lead to considerable changes for lateral distances as large as 1 nm. We showed that this behavior affects the indentation modulus with qualitative differences for various indentation radii. By using FEM simulations in which surface stresses were neglected, it was shown that indentation leads to a softening on the same length scale at the top of the step and to a hardening below the step, which explains these differences in the moduli. As stated before, this result reflects no real mechanical property of the material since this effect is highly dependent on the area and shape of the indenter.

In the case of a flatpunch, this behavior dominates the stress-induced reduction in the elastic constants, even for small tip radii. By using CR-AFM, it was not possible to measure a significant reduction of the indentation modulus close to a step of similar height outside the confidence interval. Hence, it will be an increasingly significant challenge for future measuring devices to advance to regimes that experimentally unravel the stress-induced reduction in the elastic constants. Simulations were carried out for GaN, but the results can be generalized for materials that are known to form sharp steps of comparable height.

## Supporting Information

File 1 Information about the influence of finite size effects on the indentation modulus.
