# Supplementary material for: Nanometer-resolved mechanical properties around GaN crystal surface steps
Source: Beilstein J Nanotechnol. 2014 Nov 19;5:2164–70. doi: 10.3762/bjnano.5.225 (PMC4273285; doi:10.3762/bjnano.5.225)
Supplement: File 1 — Information about the influence of finite size effects on the indentation modulus. [file Beilstein_J_Nanotechnol-05-2164-s001.pdf]

Supporting Information

for

Nanometer-resolved mechanical properties around GaN crystal surface steps

Jörg Buchwald*[1], Marina Sarmanova[2], Bernd Rauschenbach[1,2] and Stefan G. Mayr*[1,2,3]

Address: [1]Leibniz-Institut für Oberflächenmodifizierung e.V. (IOM), Permoserstr. 15, 04318 Leipzig, Germany; [2] Fakultät für Physik und Geowissenschaften, Universität Leipzig, 04103 Leipzig, Germany and [3]Translationszentrum für regenerative Medizin (TRM), Universität Leipzig, 04103 Leipzig, Germany

Email: Jörg Buchwald - joerg.buchwald@iom-leipzig.de,

Stefan G. Mayr - stefan.mayr@iom-leipzig.de

* Corresponding author

Finite size effects

Finite size effects

As stated in the text, finite size effects play a non-negligible role especially for the molecular dynamics simulations, in which the amount of atoms is restricting the size of the model domain. These result in different absolute bulk values of the indentation modulus for different contact radii. This phenomenon was studied systematically for a flatpunch indenter with contact radius $r_c = 1$ nm and an acting force of $F = 25$ nN on a cylindrical model domain, with a free cylinder barrel and a fixed cylinder base by using finite element simulations. Figure S1 shows the indentation modulus for GaN varying the height h and keeping the radius R of the model domain constant (black dots) and the other way round (red squares).

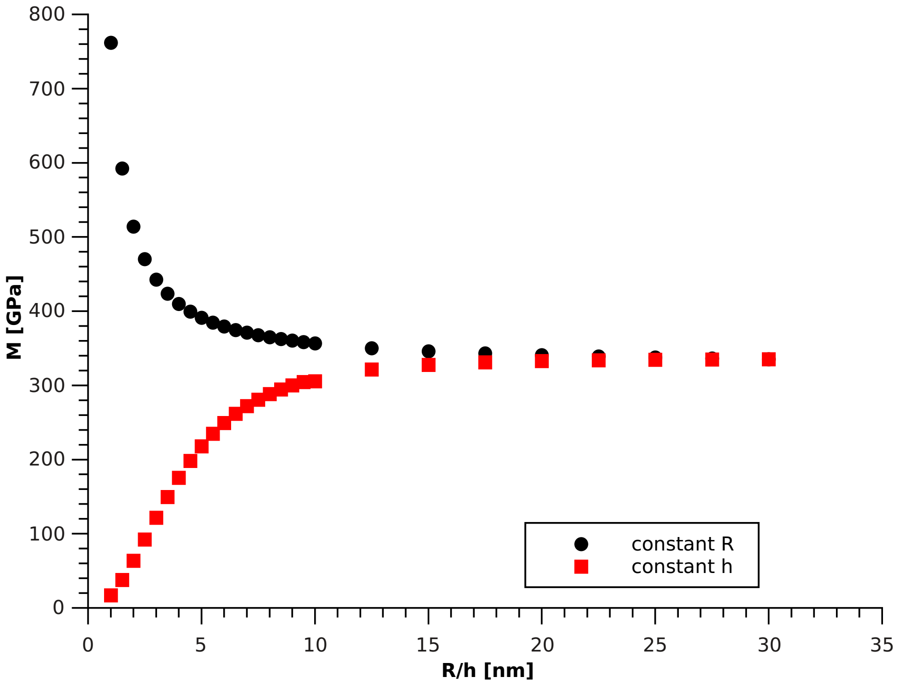

Figure S1: Changes of the indentation modulus due to finite size effects.

In summary, one can conclude from the scalability of continuum mechanics that one needs about twenty contact radii along the lateral and normal directions to get correct moduli within an error of 1%.
